# Intestinal dysbiosis and reduced immunoglobulin-coated bacteria associated with coeliac disease in children

**DOI:** 10.1186/1471-2180-10-63

**Published:** 2010-02-24

**Authors:** Giada De Palma, Inmaculada Nadal, Marcela Medina, Ester Donat, Carmen Ribes-Koninckx, Miguel Calabuig, Yolanda Sanz

**Affiliations:** 1 Ecofisiología Microbiana y Nutrición, Instituto de Agroquímica y Tecnología de Alimentos (CSIC), Apartado 73, 46100 Burjassot, Valencia, Spain; 2 Hospital Universitario La Fe, Avenida Campanar 21, 40009 Valencia, Spain; 3 Hospital General Universitario, Avenida Tres Cruces s/n 46014 Valencia, Spain

## Abstract

**Background:**

Coeliac disease is a chronic intestinal inflammatory disorder due to an aberrant immune response to dietary gluten proteins in genetically predisposed individuals. Mucosal immune response through IgA secretion constitutes a first line of defence responsible for neutralizing noxious antigens and pathogens. The aim of this study was the characterization of the relationships between immunoglobulin-coated bacteria and bacterial composition of faeces of coeliac disease (CD) patients, untreated and treated with a gluten-free diet (GFD) and healthy controls.

**Results:**

IgA-coated faecal bacterial levels were significantly lower in both untreated and treated CD patients than in healthy controls. IgG and IgM-coated bacterial levels were also significantly lower in treated CD patients than in untreated CD patients and controls. Gram-positive to Gram-negative bacteria ratio was significantly reduced in both CD patients compared to controls. *Bifidobacterium*, *Clostridium histolyticum*, *C. lituseburense *and *Faecalibacterium prausnitzii *group proportions were less abundant (*P *< 0.050) in untreated CD patients than in healthy controls. *Bacteroides-Prevotella *group proportions were more abundant (*P *< 0.050) in untreated CD patients than in controls. Levels of IgA coating the *Bacteroides-Prevotella *group were significantly reduced (*P *< 0.050) in both CD patients in comparison with healthy controls.

**Conclusions:**

In CD patients, reduced IgA-coated bacteria is associated with intestinal dysbiosis, which altogether provide new insights into the possible relationships between the gut microbiota and the host defences in this disorder.

## Background

Coeliac disease (CD) is a chronic intestinal inflammatory disorder triggered by the ingestion of gluten proteins in susceptible individuals. The active phase of the disease is characterized by a pro-inflammatory intestinal milieu resulting from an aberrant immune response to dietary gluten, along with increased epithelial permeability, which may favour the traffic of luminal antigens to the submucosa [1]. In CD patients, gliadin peptides can activate either an adaptive immune response dominated by Th_1 _pro-inflammatory cytokines (e.g. IFN-γ) within the mucosa or an innate immune response mediated by IL-15, both of which lead to epithelial cell killing [2]. Gliadin also activates the zonulin pathway leading to an increase in intestinal permeability [1].

The aetiology of CD is multifactorial, involving genetic and environmental factors. This disorder is strongly associated to the human leukocyte antigen genes (HLA). Approximately 95% of the patients inherit the alleles encoding for the HLA-DQ2 and HLA-DQ8 molecules, but only a small percentage develops CD [3]. Studies of identical twins have also shown that one twin did not develop CD in 25% of the cases studied [4], supporting the role played by environmental factors in the aetiology of this disorder. However, the elements leading to a breakdown in oral tolerance to gluten in predisposed individuals are as yet unknown. The gut microbiota constitutes a complex pool of antigens separated from the mucosal immunocompetent cells by just a single layer of epithelial cells. In this mucosal immune system IgA constitutes a first line of defence responsible for neutralizing noxious antigens and pathogens [5]. In fact, malfunction of immune cells of Peyer Patches in production of secretory IgA has been considered a risk factor for CD development [6]. It has also been speculated that a transient infection could promote inflammation and increase permeability of the mucosa to antigens by activating a Th_1 _response with secretion of IFN-γ, the major pro-inflammatory cytokine in CD patients [7,8]. Moreover, alterations in the intestinal microbiota composition of CD children in comparison with that of healthy controls, as well as changes in the metabolites derived from the gut microbial activity have been recently reported [9-12]. Nevertheless, the possible relationship between the gut microbiota composition and the first line of immune defence in CD patients remains uncharacterized.

Herein, the percentage of immunoglobulin-coated bacteria and the faecal microbiota composition of children with CD (untreated and treated with a gluten-free diet [GFD]) and controls were evaluated, thus shedding light on the possible associations between the intestinal bacteria and the host defences in this disorder.

## Results

### Immunoglobulin-coated bacteria of faeces from CD patients

**Figure 1:**
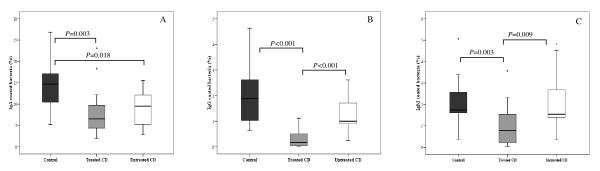
**Immunoglobulin-coated bacteria in faecal samples from untreated (white bars) and treated CD patients (grey bars) and healthy controls (black bars) as assessed by FCM**. Panel A, IgA-coated bacteria; Panel B, IgG-coated bacteria; Panel C, IgM-coated bacteria. Date are expressed as a proportion of bacterial cells labelled with FITC-F(ab')2 antihuman IgA, IgG or IgM to total cell population hybridising with propidium iodine. Median values and ranges are given. *Significant differences were established at *P *< 0.050 by applying the Mann-Whitney *U*-test.

Immunoglobulin-coated bacteria were quantified in faeces of both CD patient groups and healthy controls to establish whether CD could be associated with gut barrier defects or abnormal immune responses to the intestinal microbiota (Figure 1). Overall, higher percentages of IgA, IgM and IgG-coated bacteria were detected in healthy controls than in both CD patient groups. The proportions of IgA-coated bacteria were significantly lower in untreated (*P *= 0.018) and treated CD patients (*P *= 0.003) than in healthy controls. The proportions of IgG and IgM-coated bacteria were also significantly lower in treated CD patients than in controls (*P *< 0.001 and *P *= 0.003, respectively) and untreated CD patients (*P *< 0.001 and *P *= 0.009, respectively). The levels of IgG were also slightly lower in untreated CD patients than in healthy controls but the differences were not significant (*P *= 0.069).

### General microbiota composition of faeces from CD patients

**Figure 2:**
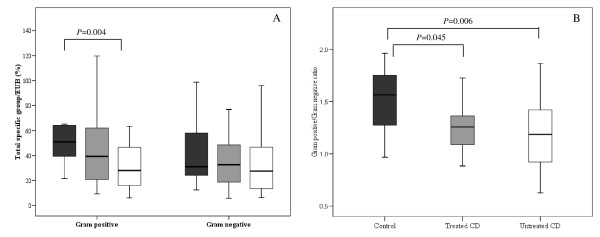
**General composition of the faecal microbiota of untreated (white bars) and treated CD patients (grey bars) and healthy controls (black bars) as assessed by FISH and FCM**. Data are expressed as proportions of bacterial cells hybridising with group-specific probes to total bacteria hybridising with EUB probe 338. Total Gram-negative bacteria and Gram-positive bacteria were calculated by adding the relative proportions of the corresponding groups detected by using group-specific probes. Median values and ranges are given. *Significant differences were established at *P *< 0.05 by applying the Mann-Whitney *U*-test.

The composition of faecal microbiota of CD patients treated and untreated with a GFD and healthy controls was characterized by using oligonucleotide probes targeting the main bacterial groups colonizing the human gut (Figure 2; Table 1). The three groups of children under study were matched by age considering the variability of the composition of human microbiota during the first years of life. Total Gram-positive bacterial populations were the highest in healthy controls and the lowest in untreated CD patients, while it reached intermediate values in treated CD. These differences were statistically significant (*P *= 0.004) between untreated CD patients and controls (Figure 2A). Gram-positive bacterial levels did not normalize completely after a long-term GFD in treated CD patients, although the differences did not reach statistical significance (*P *= 0.203) when compared with controls. Total Gram-negative bacteria reached similar values (ranging from 27.5 to 32.7%) in faeces from the three population groups (*P *= 0.323-0.650; Figure 2A). The ratio of total Gram-positive to Gram-negative bacteria was the highest in healthy controls and significantly reduced in treated CD patients (*P *= 0.045) and even more in untreated CD patients (*P *= 0.006).

**Table 1:** Faecal microbiota composition of untreated and treated CD patients and age-matched healthy controls assessed by FISH and FCM

Microbial groups	Specific group-probed cells/EUB-388 cells (%)^1^					
	
	Untreated CD (n = 24)		Treated CD (n = 18)		Control (n = 20)	
	
	Median	Range	Median	Range	Median	Range
*Bifidobacterium*	7.73	22.08-3.27	9.20	33.82-1.58	12.54	33.68-6.94
*C. histolyticum*	5.26	27.61-0.71	9.41	39.60-2.95	11.61	35.69-0.16
*C. lituseburense*	3.23	27.24-0.17	4.41	29.85-0.28	6.83	19.56-1.05
*Lactobacillus-Enterococcus*	1.94	10.93-0.14	1.12	9.30-0.22	1.76	16.47-0.25
*Staphylococcus*	10.36	37.38-0.89	16.49	42.91-0.51	18.04	41.32-0.19
*Bacteroides-Prevotella*	3.54	20.85-0.80	2.61	15.07-0.25	2.32	5.53-0.33
*E. coli*	5.20	23.42-0.48	6.39	28.77-0.55	7.32	28.26-1.10
*F. prausnitzii*	6.03	37.50-1.07	11.09	37.84-2.95	13.88	37.08-2.32
Sulphate-reducing bacteria	9.58	38.02-2.84	9.82	41.74-2.09	10.02	36.92-2.92

^1 ^Data were expressed as proportions of bacterial cells hybridising with group-specific probes to total bacteria hybridising with EUB probe 338.

* Statistical significant differences were calculated using the Mann-Whitney *U*-test and established at *P *< 0.050.

### Specific microbiota composition of faeces from CD patients

The specific group and species composition of the faecal microbiota of CD patients with untreated and treated CD and healthy controls are shown in Table 1. The highest differences in the relative abundance of specific bacterial groups were found between untreated CD patients and healthy controls, while treated CD patients generally showed intermediate values. *Bifidobacterium *proportions were significantly lower in untreated CD patients than in healthy controls (*P *= 0.009), while treated CD patients displayed intermediate values. Similarly, the relative abundance of bacteria belonging to *C. histolyticum*, *C. lituseburense *and *F. prausnitzii *groups proved to be significantly lower in untreated CD patients than in healthy subjects (*P *= 0.031, *P *= 0.024 and *P *= 0.045, respectively), whereas treated CD patients showed intermediate values. The *Bacteroides-Prevotella *group proportions were significantly more abundant in untreated CD patients than in healthy controls (*P *= 0.033). *Escherichia coli, Staphylococcus*, *Lactobacillus-Enterococcus *and sulphate-reducing bacteria reached similar proportions in the three groups of children regardless of their health status.

### Immunoglobulin A coating specific bacterial groups in faeces from CD patients

Of the total bacteria, the percentage of IgA coating *Bacteroides-Prevotella *group was significantly higher in healthy patients than in untreated CD patients (*P *= 0.014) and treated CD patients (*P *= 0.019). A 10.93% (6.13-20.13) of *Bacteroides-Prevotella *group from healthy patients was IgA-coated, while a 4.24% (4.68-6.54) and a 4.97% (0.88-8.34) was IgA-coated in untreated and treated CD patients, respectively. Accordingly, within the *Bacteroides-Prevotella *population, the percentage which was coated with IgA was significantly higher in healthy controls (69.02%; 40.54-81.61) than in untreated CD (*P *= 0.033) (25.42%; 7.09-55.09), while no differences were detected with treated CD patients. No differences were found in the proportion of IgA coating the *Bifidobacterium *group between CD patients and healthy controls. The percentage of IgA-coated *Bifidobacterium *was higher (*P *< 0.05) than that of IgA-coated *Bacteroides-Prevotella *in all groups of children.

## Discussion

This study has characterized faecal microbiology and immunoglobulin-associated features in active and non-active stages of CD in children and in age-matched controls with an aim to furthering our understanding of the interplay between the gut microbiota and the host defences in this disorder. Immunoglobulin secretions constitute a primary line of defence of the mucosal surface against noxious antigens and pathogens, and contribute to the intestinal homeostasis preventing clinical inflammation. The colon predominantly harbours IgA-secreting plasma cells (90%); moreover, 4% cells secrete IgG and 6% cells secrete IgM. A considerable percentage of faecal bacteria was coated with IgA (14.71%) in healthy individuals, whereas a lower bacterial percentage was coated with IgG and IgM, and a similar trend was observed in CD patients, as reported in other subjects [13]. Significantly lower levels of IgA-coated bacteria were detected in faecal samples of untreated and treated CD patients when compared to healthy controls. It can be speculate that these results could reflect the existence of a barrier defect in CD patients, which fails to stabilise the gut microbiota and prevent the host from the invasion of harmful antigens and pathogens. In addition, treated CD patients showed lower levels of IgG and IgM coated bacteria. In contrast, IBD patients displayed a higher percentage of immunoglobulin-coated faecal bacteria in active disease and shortly after remission, supporting the concept that the mucosal tolerance to the gut microbiota is deregulated in these patients [5].

A remarkable reduction in Gram-positive bacterial populations was characteristic of the active phase of the disease while its abundance was partially restored in patients under a GFD. In addition, a reduction in the ratio of Gram-positive to Gram-negative bacteria was found in the patients regardless of the phase of the disorder. The levels of total Gram-positive bacteria were also lower in duodenal biopsies of patients with active and inactive CD than in controls, while the proportions of total Gram-negative bacteria were over-represented particularly in biopsies of active CD patients [12]. Therefore, the results obtained first in biopsies and now in faeces from children of the same age confirm similar structural changes in the composition of the gut microbiota associated with CD. The reductions in beneficial Gram-positive bacteria could favour the residence and interactions of harmful Gram-negative bacteria within the mucosal surface of CD patients, thereby contributing to loss of gluten tolerance. Antigenic structures of Gram-negative bacteria such as flagellins and lipopolysaccharides have been related to the inflammatory responses and pathogenesis of IBD [14]. Shifts in the intestinal microbiota, characterized by increases in pro-inflammatory Gram-negative bacteria, have also been shown to aggravate murine colitis via activation of acute inflammation through Toll-like receptor signalling [15].

Of the specific bacterial groups analysed, the *Bifidobacterium *population was significantly reduced in faecal samples of untreated CD patients as compared with controls. *Bifidobacterium *populations significantly decreased or slightly decreased in faeces of IBD patients, as detected by cultural techniques and real time PCR, respectively [16]. The benefits obtained by administering some *Bifidobacterium *strains as part of probiotic mixtures or symbiotics (probiotics combined with prebiotics) in ulcerative colitis and pouchitis also support the notion that this bacterial group is relevant to IBD [17]. *C. histolyticum, C. lituseburense *and *F. prausnitzii *groups were present in higher proportions in healthy individuals than in CD patients; particularly, the abundance of *C. histolyticum *followed a similar trend to that found in biopsy specimens although the differences were not significant [12]. *C. coccoides *and *C. leptum *groups were lower in faeces of Crohn's disease and ulcerative colitis patients when determined by real-time PCR [16]. A depletion of *F. prausnitzii *population in faecal mucus of active Crohn's disease, but not in ulcerative colitis, has also been detected [18]. Comparative analysis of biopsy and faecal samples of IBD patients, based on genomic-library sequencing analysis, also showed reductions in *Firmicutes *belonging to the class *Clostridia *in active and in remission Crohn's disease patients as compared to healthy or ulcerative colitis groups [19,20]. Although some studies are controversial, it appears that the presence of certain *Clostridium *groups and *F. prausnitzii *is deficient in luminal or mucosa-associated microbiotas of Crohn's disease and probably of CD patients too. These components of the microbiota are producers of butyrate, which is an important energy source for colonocytes and exerts anti-inflammatory effects, for instance by inhibiting the lipopolysaccharide-induced cytokine response [19]. In contrast, the *Bacteroides-Prevotella *group was found in higher proportions in untreated CD patients than in controls, as previously detected in duodenal biopsy specimens [12]. Associations between the phylum *Bacteroidetes *and Crohn's disease were revealed by comparative bacteriological analysis of biopsy specimens of Crohn's disease and ulcerative colitis patients by denaturing gradient gel electrophoresis (DGGE) [17]. Similar comparative analyses of the mucosal-associated microbiota by genomic-library sequencing of 16S rRNA genes showed increases in *Proteobacteria *and *Bacteroidetes*, particularly in Crohn's disease patients [19]. Nevertheless, a recent study reported that *B. fragilis *and *B. vulgatus *were found at lower levels in faeces of IBD patients when compared to those of healthy controls [16].

As *Bacteroides *and *Bifidobacterium *seem to be possible relevant bacterial groups to CD, specific percentages of IgA coating these two bacterial groups were also determined. Interestingly, the proportions of IgA-coated *Bacteroides-Prevotella *were higher in healthy individuals than in treated and untreated CD patients, suggesting an increased defensive response of the gut mucosal immune system to this bacterial group in healthy children than in CD patients. The combination of an increased proportion of *Bacteroides-Prevotella *group in faecal samples of CD patients together with a weaker defensive IgA response could explain the recurrent relationship found between *Bacteroides *and inflamed gut mucosa in CD [12,21], although more direct evidence is needed to confirm this hypothesis. A higher percentage of IgA-coated *Bifidobacterium *than IgA-coated *Bacteroides-Prevotella *was detected in all groups of children, similarly to other studies [5]. This could be a consequence of an increased interaction between the gut mucosal immune system and this bacterial group [5], which contributes to mucosal tolerance towards high gut *Bifidobacterium *concentrations.

## Conclusions

This study confirms that in CD patients there is an alteration in the type of faecal immunoglobulin-coated bacteria that is associated with a shift in the structure of the microbiota. In particular, increases in the relative abundance of *Bacteroides-Prevotella *group are paralleled to reductions in the IgA coating this group, which could suggest a reduction of of the host defences against this bacterial group. However, the possible clinical consequences of these finding are still unknown and their elucidation would require further investigations.

## Methods

### Subjects

Altogether 62 children were included in the study: 24 untreated CD patients (mean age 5.5 years, range 2.1-12.0 years) on a normal-gluten containing diet, showing clinical symptoms and signs of the disease, positive CD serology markers (anti-gliadin antibodies and anti-transglutaminase antibodies) and signs of severe enteropathy by duodenal biopsy examination classified as type 3 according to Marsh classification of CD; 18 treated CD patients (mean age 5.5 years, range 1.0-12.3 years) on a gluten-free diet for at least 2 years, without symptoms of the disease, showing negative CD serology markers and normal mucosa architecture; and 20 healthy children (mean age 5.3 years, range 1.8-10.8 years) without known gluten intolerance.

None of the children were treated with antibiotics at least 1 month before to the faecal sampling. The study was conducted in accordance with the ethical rules of the Helsinki Declaration (Hong Kong revision, September 1989), following the EEC Good Clinical Practice guidelines (document 111/3976/88 of July 1990) and current Spanish law, which regulates clinical research in humans (Royal Decree 561/1993 regarding clinical trials). Children were enrolled in the study after written informed consent obtained from their parents.

### Faecal sample preparation

Faeces from the three groups of children were collected in sterile plastic boxes, frozen immediately after collection at -20°C, and stored until analysed. Faeces were diluted 1: 10 (w/v) in PBS (pH 7.2) and homogenized in a Lab Blender 400 stomacher (Seward Medical London, UK) for 5 min. After low-speed centrifugation (2,000 *g*, 2 min), the supernatant was collected. For bacterial quantification, cells were fixed by adding 4% paraformaldehyde solution (Sigma, St Louis, MO) and incubated overnight at 4°C. After fixation, bacteria were washed twice in PBS by centrifugation (13,400 *g *for 5 min). Finally, cell pellets were suspended in a PBS/ethanol mixture (1:1) and stored at -80°C until analyzed as previously described [12].

### Immunoglobulin-coated bacterial analysis

Bacterial cells from 20 μl of the supernatant obtained after low-speed centrifugation were collected (12,000 rpm for 5 min). The pellet was resuspended in 60 μl 1% (w/v) BSA/PBS, containing 1% (v/v) FITC-labelled F(ab')2 antihuman IgA, IgG or IgM (CALTAG Laboratories, Burlingame, CA). Another aliquot of each sample was pelleted and resuspended in 60 μl 1% (w/v) BSA/PBS and used as control. After 30 min incubation, suspensions were washed twice with PBS. Bacterial pellet was finally resuspended in 500 μl PBS and mixed with 20 μl propidium iodine (100 mg l^-1^) to label total bacteria before flow cytometry detection [5]. To determine the percentage of IgA coating the *Bacteroides-Prevotella *and *Bifidobacterium *groups, the hybridised bacteria were resuspended in 60 μl 1% (w/v) BSA/PBS, containing 1% (v/v) FITC-labelled F(ab')2 antihuman IgA (CALTAG Laboratories, Burlingame, CA). After 30 min incubation, suspensions were washed twice with cold PBS, stored at 4°C in the dark and analysed within few hours, as previously described [5].

### Microbiological analysis by fluorescent *in situ *hybridisation

The bacterial groups present in faeces were quantified by fluorescent in *situ *hybridization (FISH) using group-specific probes (MOLBIOL, Berlin, Germany). The specific probes and controls used in this study, as well as the hybridization conditions, are shown in Table 2. In the case of *E. coli *a 50°C hybridization temperature was used. The EUB 338 probe, targeting a conserved region within the bacterial domain, was used as a positive control [22] and the NON 338 probe was used as a negative control to eliminate background fluorescence [23]. Control probes were covalently linked at their 5' end either to indocyanine dye Cy3 or to fluorescein isothiocyanate (FITC). Specific cell enumeration was performed by combining each of the group-specific FITC-probes with the EUB 338-Cy3 probe as previously described [12]. Briefly, fixed cell suspensions were incubated in the hybridization solution (10 mmol l^-1 ^Tris-HCl, 0.9 mol l^-1 ^NaCl pH 8.0 and 10% SDS) containing 4 ng μl^-1 ^of each fluorescent probe at appropriate temperatures, overnight. Then, hybridised cells were pelleted by centrifugation (12,000 rpm for 5 min) and resuspended in 500 μl PBS solution for flow-cytometry analysis.

**Table 2:** Oligonucleotide probes and hybridisation conditions used in the analysis of faecal bacteria by FISH and FCM

Probe	Target Bacterial group	Sequence (5'-3')	Hybridisation Conditions (°C)	References
Eub 338	Domain bacteria	GCT GCC TCC CGT AGG AGT	50	[13]
Non 338	Negative control	ACA TCC TAC GGG AGG C	50	[14]
Bif 164	*Bifidobacterium*	CAT CCG GCA TTA CCA CCC	50	[24]
Lab 158	*Lactobacillus/Enterococcus*	GGT ATT AGC A(C/T)C TGT TTC CA	45	[25]
Bac 303	*Bacteroides/Prevotella*	CCA ATG TGG GGG ACC TT	45	[26]
Ecol 1513	*Escherichia coli*	CAC CGT AGT GCC TCG TCA TCA	50	[27]
Chis 150	*Clostridium histolyticum*	TTA TGC GGT ATT AAT CT(C/T) CCT TT	50	[28]
C Lis 135	*Clostridium lituseburense*	GTT ATC CGT GTG TAC AGG G	50	[28]
FPrau 645	*Faecalibacterium prausnitzii*	CCT CTG CAC TAC TCA AGA AAA AC	50	[29]
SRB 687	Sulphate-reducing bacteria	TAC GGA TTT CAC TCC T	50	[30]
STA	*Staphylococcus*	TCC TCC ATA TCT CTG CGC	50	[31]

### Flow cytometry

Flow cytometry detections were performed using an EPICS^® ^XL-MCL flow cytometer (Beckman Coulter, Florida, USA) as previously described [12]. This instrument is equipped with two light scatter detectors that measure forward (FSC) and side scatter (SSC) and fluorescence detectors that detect appropriately filtered light at green (FL1, 525 nm) and red-orange (FL3, 620 nm) wavelengths. The event rate was kept at the lowest setting (200-300 events per second) to avoid cell coincidence. A total of 15,000 events were recorded in a list mode file and analyzed with the System II V.3 software (Beckman Coulter). The proportion of each bacterial group was expressed as a ratio of cells hybridising with the FITC-labelled specific probe to cells hybridising with the universal EUB 338-Cy3 probe [12]. Total Gram-negative bacteria and Gram-positive bacteria were calculated by adding the relative proportions (%specific group/EUB) of the corresponding groups. Immunoglobulin-coated bacteria was expressed as a ratio of bacterial cells labelled with FITC-labelled F(ab')2 antihuman IgA, IgG or IgM to the bacterial cell populations hybridising with either propidium iodine, EUB338 probe, *Bacteroides-Prevotella *group-specific probe or *Bifidobacterium *group-specific probe [5].

### Statistical analyses

Statistical analyses were done using the SPSS 11.0 software (SPSS Inc, Chicago, IL, USA). Due to non-normal distribution, microbial and immunoglobulin coating bacterial data are expressed as medians and ranges (maximum-minimum values). The differences between two groups of samples were determined by applying the Mann-Whitney *U *test. In every case, a *P*-value < 0.05 was considered statistically significant.

## Abbreviations

CD: Coeliac disease; Ig: Immunoglobulin; HLA: Human Leukocyte Antigen; FCM: Flow cytometry; FISH: Fluorescence *in situ *hybridization; GFD: Gluten-free diet; IBD: Inflammatory bowel disease.

## Authors' contributions

GDP, IN and MM carried out the microbiological and immunoglobulin analyses, ED, CRK and MC participated in the recruitment and clinical examination of the studied children. YS conceived of the study and draft the manuscript. All authors read and approved the final version of the manuscript.
